# The Effect of Dry Needling on Sleep Quality in Individuals With Musculoskeletal Pain Disorders

**DOI:** 10.1155/prm/7008601

**Published:** 2026-02-26

**Authors:** Ray M. Lunasin, Guy Terry, Sharon Wang-Price

**Affiliations:** ^1^ Department of Orthopedic Surgery, Mayo Clinic, Rochester, Minnesota, USA, mayo.edu; ^2^ Department of Physical Medicine & Rehabilitation, Mayo Clinic, Rochester, Minnesota, USA, mayo.edu; ^3^ Program in Physical Therapy, Mayo Clinic School of Health Sciences, Mayo Clinic College of Medicine and Science, Rochester, Minnesota, USA, mayoclinic.org; ^4^ School of Physical Therapy, Texas Woman’s University, Dallas, Texas, USA, twu.edu

**Keywords:** dry needling, musculoskeletal, outcome measures, pain, sleep disturbance

## Abstract

**Objectives:**

To explore existing evidence and identify whether dry needling (DN) intervention has effects on sleep disturbance in patients with musculoskeletal pain.

**Methods:**

The Arksey and O’Malley framework guided the scoping review methodology. Seven databases were searched for clinical trials investigating DN in musculoskeletal pain disorders. Methodological quality was assessed using the Physiotherapy Evidence Database (PEDro) scale. Data extraction included study year, location, study design, musculoskeletal pain disorder, needling intervention type, and sleep outcome measure utilized.

**Results:**

After duplicates were removed, 2292 articles were identified, and 33 studies were included in the review after independent screening. Two supplemental searches (May 2023 and January 2024) in addition to a hand search yielded an additional 146 articles. A total of 21 of those studies were also included, increasing the total to 54 studies. A total of 46 (84%) articles were RCTs and 8 (16%) were single‐group, pretest–posttest clinical trials. A total of 9 studies were of optimal quality (PEDro score ≥ 8), and 11 were of moderate‐to‐high quality (PEDro score = 7).

**Discussion:**

Due to significant variability in intervention, sleep outcome measurement, patient population, and study methodology quality, the evidence is mixed and inconclusive to support or refute the effects of DN on sleep deficits in individuals with musculoskeletal pain. However, future studies investigating the effects of needling interventions on musculoskeletal pain conditions should include valid sleep outcome measurements if considered.

## 1. Introduction

Chronic pain is a prevalent debilitating condition that affects daily work and life for many adults in the United States. As of 2021, an estimated 51.6 million American adults (20.9%) experienced chronic pain [1]. Recommendations from current clinical practice guidelines advise nonpharmacological treatment as frontline care for pain management in musculoskeletal conditions [2]. Among nonpharmacological treatment options, dry needling (DN) has been demonstrated to be an effective intervention in addressing chronic musculoskeletal pain [3, 4, 5]. DN is a minimally invasive technique targeting myofascial trigger points to alleviate pain and restore function [3]. DN has been applied across various musculoskeletal conditions, including chronic neck pain, low back pain, orofacial pain disorders, and peripheral joint conditions such as knee osteoarthritis, hip pain, and Achilles tendinopathy. Recent evidence suggests DN may also benefit individuals with temporomandibular disorders and headache syndromes, highlighting its versatility in clinical practice [3, 4, 6]. Although DN and acupuncture share the use of filiform needles, they differ fundamentally in rationale and anatomical targets. DN focuses on myofascial trigger points based on neuromusculoskeletal principles, whereas acupuncture is rooted in traditional Chinese medicine and targets meridian points [5, 7].

Although DN has been observed as an effective treatment option for chronic pain, understanding its physiological mechanisms of effect is still underway. Research has shown a bidirectional relationship between pain and sleep. Persistent and chronic pain has been shown to disrupt sleep, while short or disturbed sleep increases spontaneous pain, thus negatively affecting pain perception [7, 8, 9, 10]. Epidemiological evidence has also demonstrated that poor sleep quality and insufficient sleep duration are risk factors for the development of chronic pain [11, 12].

Despite the well‐established bidirectional relationship between impaired sleep and pain, the neurobiological mechanisms underlying this reciprocal relationship remain scarce. Nevertheless, several mechanisms have been proposed to explain the association between sleep and pain. First, studies have shown that sleep dysfunction directly affects the immune system, facilitating a glia‐mediated proinflammatory state and then resulting in hyperalgesia [9, 13, 14]. The facilitation of pain processes due to sleep dysfunction could explain the bidirectional relationship between chronic pain and sleep dysfunction. Additionally, both chronic pain and sleep dysfunction have been shown to facilitate neurons in the monoaminergic pathways, which increase the activity of neurotransmitters, including dopamine, serotonin, and norepinephrine [15, 16, 17, 18]. These neurotransmitters regulate both the sleep–wake system and endogenous pain substances, such as melatonin, nitric oxide, orexin, and vitamin D [15, 17, 18]. Additionally, central structures such as the hypothalamic–pituitary–adrenal (HPA) axis, thalamus, and cortical regions are involved in both sleep regulation and chronic pain [15, 17, 18]. Sleep generally exerts an inhibitory effect on the HPA axis, but under certain physiological conditions, it can activate the axis to maintain cortisol secretion necessary for energy homeostasis [19]. This bidirectional interaction emphasizes the complexity of neuroendocrine regulation in sleep and pain. When HPA axis activity is heightened, it may lead to increased arousal and disrupted sleep patterns [19, 20]. Alternatively, reduced HPA function has been observed in individuals with chronic multisite musculoskeletal pain disorders, further highlighting the intricate interplay between these systems [21].

The prevalence of sleep disturbance in individuals with chronic pain has been estimated to be between 50% and 80% [7]. Despite the high prevalence of sleep disturbance in patients with chronic pain, evidence of conservative interventions effectively addressing both sleep dysfunction and pain remains limited [9, 22, 23, 24]. DN also has the potential of being an effective option for addressing sleep, with its analgesic effects theorized to be neurophysiological [25, 26], as DN has been shown to reduce peripheral nociception, decrease spinal dorsal horn neuron activity, and increase the activation of the sensorimotor cortical network [27, 28]. These neurophysiological changes are mediated by biochemical substances, such as Substance P, bradykinin, norepinephrine, calcitonin gene–related peptide (CGRP), interleukin‐1 *β* (IL1), and tumor necrosis factor *α* (TNF), which also have been shown to mediate sleep in addition to modulating pain. Significantly higher concentration levels of these biochemical substances have also been observed in individuals with sleep disturbance [29, 30, 31, 32]. Furthermore, DN has been shown to increase the number of endogenous opioids peripherally available via the endocannabinoid system [33, 34, 35, 36]. Therefore, DN has the potential to improve sleep dysfunction in individuals with pain through biochemical substances associated with both pain modulation and sleep regulation.

Because the available evidence regarding the effects of DN on sleep disturbance is limited, a scoping review is warranted. Therefore, this scoping review aimed to explore existing evidence and identify whether needling intervention affects sleep disturbance in patients with musculoskeletal pain.

## 2. Methodology

### 2.1. Framework, Protocol, and Registration

A systematic scoping review methodology was selected after a preliminary literature search produced a limited number of studies regarding the research question. The 5‐step methodological framework for a scoping review proposed by Arksey and O’Malley [37, 38] was used to guide this scoping review. In addition, this scoping review was reported according to the PRISMA Extension for Scoping Reviews (PRISMA‐ScR) [39] and was registered a priori with Open Science Framework (OSF) [40].

### 2.2. Search

Relevant articles were identified in a search of 7 online databases, including PubMed, CINAHL, SPORTDiscus, PEDro, ProQuest, ScienceDirect, and Taylor & Francis, and 2 online registries, Scopus and Web of Science, from inception to June 21, 2022, based on their relevance to the topic of interest. In addition to database searches, a preliminary grey literature search was conducted to identify sources not indexed in traditional databases. This included books, conference proceedings, and professional reports related to DN and sleep disturbance. Grey literature findings were used only to inform background understanding and were not included in the final synthesis. The search string and search terms (Table 1) were developed in consultation with a health science librarian. The inclusion criteria included the following: (1) English language, (2) human subjects, and (3) clinical trial study design. The exclusion criteria included the following: (1) nonmusculoskeletal disorders, (2) non‐needling intervention, (3) wet needling or injection, and (4) secondary research sources. Eligibility criteria included clinical trials from inception to December 2023 investigating symptomatic subjects with musculoskeletal pain, receiving needling intervention, and recording sleep outcome measurements. Trials investigating injection therapy, nonmusculoskeletal pain diagnoses, or asymptomatic populations were excluded.

**TABLE 1:** Search string and search terms.

Search string	(“Musculoskeletal Pain” OR “musculoskeletal disorders” OR “musculoskeletal injuries” OR “musculoskeletal injury” OR “musculoskeletal system” OR “Musculoskeletal Abnormalities” OR “myalgia” OR “cumulative trauma disorders” OR “chronic pain” OR “Myofascial Pain Syndromes” OR “Myofascial Trigger Point Pain” OR “Muscle Pain” OR “Repetitive motion Disorders”) AND (“Dry Needling” OR “trigger point needling” OR “dry needling technique” OR “acupuncture” OR “acupuncture therapy” OR “trigger point acupuncture” OR “trigger point therapy” OR “intramuscular acupuncture” OR “acupuncture Analgesia” OR “Myofascial Acupuncture”) AND (“Sleep” OR “Sleep Quality” OR “Sleep Qualities” OR “Sleep Hygiene” OR “Sleep Disturbance” OR “Sleep Wake Disorder” OR “Sleep Wake Disorders”)	
Concept keyword	Synonyms	MeSH terms

Musculoskeletal Pain	“musculoskeletal disorders” “musculoskeletal injuries” “musculoskeletal injury” “musculoskeletal system” “Musculoskeletal Abnormalities” “myalgia” “cumulative trauma disorders” “chronic pain” “Myofascial Pain Syndromes” “Myofascial Trigger Point Pain” “Muscle Pain” “Repetitive motion Disorders”	“Musculoskeletal Pain” [Mesh] “Cumulative Trauma Disorders” [Mesh] “Musculoskeletal System” [Mesh] “chronic pain” [Mesh] “myalgia” [Mesh] “Myofascial Pain Syndromes” [Mesh]

Dry Needling	“trigger point needling” “dry needling technique” acupuncture “acupuncture therapy” “trigger point acupuncture” “trigger point therapy” “intramuscular acupuncture” “Acupuncture Analgesia” “Tendinomuscular Acupuncture” “Myofascial Acupuncture”	“Dry Needling”[Mesh] “Acupuncture” [Mesh] “Acupuncture Therapy” [Mesh] “Acupuncture Analgesia”[Mesh]

Sleep (Quality or Disturbance)	“Sleep qualities” “Sleep hygiene” “Sleep wake disorders” “Sleep wake disorder”	“Sleep quality”[Mesh] “Sleep hygiene” [Mesh] “Sleep wake disorders” [Mesh] “Sleep”[Mesh]

*Note:*​ PubMed recommends against using truncation or wildcards so they were not included in this search strategy.

After, determination of included studies was completed, a hand search was conducted by the primary author (R.L.), reviewing all references of the hallmark articles in addition to cross‐referencing all research literature they were cited by. The author utilized Google Scholar for all cross‐referencing of the hallmark articles. The same inclusion and exclusion criteria were applied to the hand search. Two additional searches were conducted (May 3, 2023, and Jan 17, 2024) to ensure no articles from the past year were incidentally omitted for this scoping review.

### 2.3. Study Selection

Search results were organized using Microsoft Excel (Microsoft Corporation, Redmond, WA), and citations were uploaded onto Rayyan QCRI—Intelligent Systematic Review for article screening [41]. First, the primary author (R.L.) removed the duplicates and performed a preliminary screening of titles and abstracts using the predetermined inclusion and exclusion criteria. Next, a blinded screening of titles and abstracts was conducted for exclusion by two independent reviewers (R.L. and G.T.) on Rayyan. Any articles that were not agreed upon between the two reviewers were advanced to the full‐text screening for inclusion. A blinded full‐text screening was then performed independently by the same two reviewers. However, any disagreements in article inclusion were resolved by a third reviewer (S.W.). The exact procedures were repeated after both the secondary search and the hand search.

### 2.4. Quality Assessment

Included articles that were randomized clinical trials (RCTs) were assessed for methodological quality prior to data charting using the Physiotherapy Evidence Database (PEDro) scale [42, 43], as the PEDro scale has been shown to be reliable and valid [44, 45, 46]. Additionally, the PEDro scale assists readers to quickly assess whether a clinical trial presents reliable and meaningful results for use in clinical practice [47]. The PEDro database classifies clinical trials only and scores them by adding the number of items on the scale (0–10) that have been met. Certified PEDro scores were used for RCTs listed on the PEDro website. For the articles that did not receive a score on the PEDro database, independent appraisals of these articles were completed by two reviewers (R.L. and S.W.). A trial’s quality was considered “poor” with a PEDro score of 0–3, “fair” with a score of 4–5, “good” with a score of 6–8, and “excellent” with a score of 9–10 [48]. However, studies with a score of 7 are considered moderate‐to‐high quality, whereas a score of ≥ 8 is considered optimal quality [42, 48]. For the purpose of this scoping review, if a sufficient number of RCTs were included in the review, the studies with a PEDro score ≥ 7 would be included in discussion, although the PEDro scores were reported for all studies.

### 2.5. Data Charting and Synthesis

For all included studies, the primary author (R.L.) extracted data into the following categories: study year, location, and study design; participant age, sex, musculoskeletal pain disorder, needling intervention type, and sleep outcome measure utilized. Study details, sample characteristics, intervention details, and effects on sleep were described and numerically summarized.

## 3. Results

**FIGURE 1:**
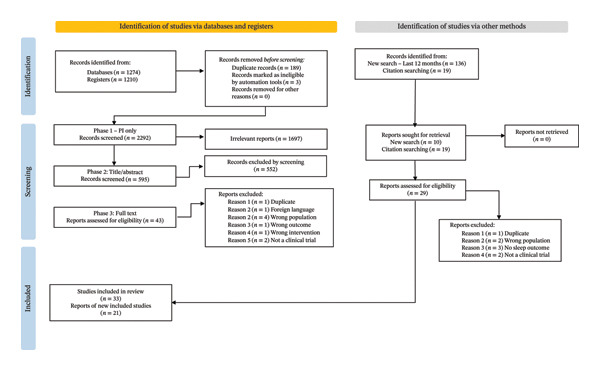
PRISMA flow diagram for database search and article screen.

After duplicates were removed, 2292 articles were identified, and 33 studies were included in the review after independent screening. Figure 1 displays the PRISM flow diagram for an overview of the database search and article screening process. The supplemental searches and the hand search yielded 14 articles being identified, with 21 studies being included, resulting in a total of 54 studies.

### 3.1. Study Characteristics

Among the 54 included studies, 46 (85%) were RCTs and 8 (15%) were single‐group, pretest–posttest clinical trials. The RCTs were published between 1992 and 2023, conducted in North America, South America, Continental Europe, Scandinavia, the Middle East, the United Kingdom, and East Asia. The remaining studies were published between 1998 and 2021, conducted in North America, Continental Europe, Scandinavia, and Southeast Asia. As the PEDro scores of 42 out of the 46 RCTs were available on the PEDro website, only 4 RCTs required independent appraisals [49, 50, 51, 52]. A consensus was reached for these 4 RCTs by the two reviewers, and a third reviewer was not required. PEDro scores of these 46 RCTs are listed in Table 2, with 3 of poor quality, 18 of fair quality, 23 of good quality, and 2 of excellent quality. Tables 3 and 4 summarize the findings of 9 optimal quality studies (PEDro score ≥ 8) and 11 moderate‐to‐high–quality studies (PEDro score = 7), respectively. The 8 non‐RCT articles did not meet the study design criteria to administer PEDro scoring. The findings of these 8 articles are summarized in Table 5.

**TABLE 2:** PEDro scores for randomized clinical trials.

PEDro score	Research studies by author	Year published
3 (*n* = 3)	Ahmed et al. [53] Ghoname et al. [54] Hamza et al. [55]	2004 1999 1999

4 (*n* = 5)	Casanueva et al. [56] Chong et al. [57] Ghoname et al. [58] Piexoto et al. [59] Razavi et al. [60]	2014 2018 1999 2021 2004

5 (*n* = 13)	Ghoname et al. [61] Gu et al. [62] Hansson et al. [63] Krusche‐Mandl et al. [64] Minakawa et al. [65] Perez‐Palomares et al. [66] Raissi et al. [67]Sator‐Katzenschlager et al. [68] Sator‐Katzenschlager et al. [69] White et al. [71] Ugurlu et al. [70] White et al. [52] Yang et al. [72]	1999 2019 2007 2019 2022 2017 2003 2010 2004 2000 2017 2001 2021

6 (*n* = 5)	Assefi et al. [73] Deluze et al. [74] Garner et al. [75] Moreira et al. [76] Serritella et al. [77]	2005 1992 2018 2023 2021

7 (*n* = 11)	Carlsson et al. [78] Dalewski et al. [79] He et al. [80] Ho et al. [81] Huang et al. [50] Karatay et al. [82] Moura et al. [83] Soderberg et al. [84] Tezel et al. [85] Weiner et al. [86] Yang et al. [87]	2001 2019 2005 2013 2022 2018 2022 2011 2019 2003 2023

8 (*n* = 7)	Castro‐Sanchez et al. [88] Couto et al. [89] Epstein et al. [49] Hadianfard et al. [90] Lara‐Palomo et al. [91] Martin et al. [92] Stieven et al. [93]	2019 2014 2023 2012 2023 2006 2020

9 (*n* = 2)	Kao et al. [51] Wheeler et al. [94]	2022 2022

**TABLE 3:** Summary of optimal quality studies (PEDro score ≥ 8).

Author	Year	Country	Patient population (*n* = )	Needling intervention	Frequency	Comparison intervention	Sleep outcome measure
Castro‐Sanchez et al. [**88**]	**2019**	**Spain**	**Fibromyalgia syndrome (*n* = 64)**	**Dry needling**	**1x/week 4 weeks**	**Myofascial release**	**PSQI**
Couto et al. [**89**]	**2014**	**Brazil**	**Myofascial pain syndrome (*n* = 78)**	**Deep intramuscular stimulation**	**2x/week 4 weeks**	**Trigger point injection and sham treatment**	**VASQS**
Epstein et al. [**49**]	**2023**	**USA**	**Advanced cancer with musculoskeletal pain (*n* = 298)**	**Acupuncture**	**1x/week 10 weeks**	**Massage therapy**	**ISI**
Hadianford et al. [90]	2012	Iran	Fibromyalgia syndrome (*n* = 30)	Acupuncture	3x/week 2 weeks	Fluoxetine	FIQ
Kao et al. [51]	2022	Taiwan	Blunt chest trauma (*n* = 72)	Press‐tack acupuncture	One session	Sham treatment	VSHSS
Lara‐Palomo et al. [**91**]	**2023**	**Spain**	**Chronic low back pain (*n* = 64)**	**Dry needling with electrical stimulation**	**1x/week 6 weeks**	**Noninvasive multicomponent intervention**	**PSQI**
Martin et al. [92]	2006	USA	Fibromyalgia syndrome (*n* = 50)	Acupuncture	6 sessions over 2–3 weeks	Sham treatment	FIQ
Stieven et al. [93]	2020	Brazil	Chronic neck pain (*n* = 116)	Dry needling + guideline‐based physical therapy	4–6 sessions over 4 weeks	Guideline‐based physical therapy only	PSQI
Wheeler et al. [**94**]	**2022**	**UK**	**Chronic plantar fasciitis (*n* = 90)**	**Dry needling**	**One session**	**Autologous blood injection with dry needling**	**PSQI**

*Note:* The bold rows indicate a positive response, and nonbold rows indicate no change.

Abbreviations: FIQ = Fibromyalgia Impact Questionnaire, ISI = Insomnia Severity Index, PSQI = Pittsburgh Sleep Quality Index, VASQS = Visual Analog Sleep Quality Scale, VSHSS = Verran Snyder–Halpern Sleep Scale.

**TABLE 4:** Summary of moderate‐to‐high‐quality studies (PEDro scores = 7).

Author	Year	Country	Patient population (*n* = )	Needling intervention	Frequency	Comparison intervention	Sleep outcome measure
Carlsson et al. [**78**]	**2001**	**Sweden**	**Chronic low back pain (*n* = 50)**	**Acupuncture**	**1x/week 8 weeks**	**Electroacupuncture or active placebo**	**VAS**
Dalewski et al. [**79**]	**2019**	**Poland**	**Chronic orofacial pain (*n* = 90)**	**Dry needling**	**1x/week 3 weeks**	**Splint therapy or NSAIDs**	**SPAQ**
He et al. [**80**]	**2005**	**Norway**	**Chronic neck and shoulder pain (*n* = 24)**	**Acupuncture**	**10 sessions over 3–4 weeks**	**Sham treatment**	**Likert scale**
Ho et al. [81]	2013	Taiwan	Acute inpatient rib fracture (*n* = 58)	Conventional oral analgesics + acupuncture	1x/day 3 consecutive days	Conventional oral analgesics + thumbtack intradermal needles	NRS
Huang et al. [**50**]	**2022**	**China**	**Chronic neck pain (*n* = 60)**	**Fu’s subcutaneous needling**	**3 sessions over 4 days**	**TENS**	**PSQI**
Karatay et al. [**82**]	**2018**	**Turkey**	**Fibromyalgia syndrome (*n* = 75)**	**Acupuncture**	**2x/week 4 weeks**	**Sham treatment or simulated acupuncture**	**NHP**
Moura et al. [**83**]	**2022**	**Brazil**	**Chronic back pain (*n* = 198)**	**Ear acupuncture only**	**1x/week 5 weeks**	**Ear acupuncture plus dry cupping**	**BPI**
Soderberg et al. [**84**]	**2011**	**Sweden**	**Chronic tension type headache (*n* = 88)**	**Acupuncture**	**10-12 sessions over 10–12 weeks**	**Relaxation training or physical training**	**MSEP**
Tezel et al. [85]	2019	Turkey	Carpal tunnel syndrome (*n* = 51)	Acupuncture + night splinting	2x/week 5 weeks	Night splinting only	NHP
Weiner et al. [86]	2003	USA	Chronic low back pain (*n* = 34)	PENS + physical therapy	2x/week 6 weeks	Sham‐PENS + physical therapy	PSQI
Yang et al. [**87**]	**2023**	**USA**	**Chronic pain (*n* = 268)**	**Auricular acupuncture**	**1x/week 10 weeks**	**Electroacupuncture or usual care**	**PSQI**

*Note:* The bold rows indicate a positive response, and nonbold rows indicate no change.

Abbreviations: BPI = Brief Pain Inventory, MSEP = Minor Symptom Evaluation Profile, NHP = Nottingham Health Profile, NRS = Numerical Rating Scale, PENS = percutaneous electrical nerve stimulation, PSQI = Pittsburgh Sleep Quality Index, SPAQ = Sleep and Pain Activity Questionnaire, TENS = transcutaneous electrical nerve stimulation, VAS = visual analog scale.

**TABLE 5:** List of non‐RCT studies.

Author	Year published	Population	Needling intervention	Sleep outcome measure	Effect of intervention
Borg‐Stein et al. [95]	2003	Low back pain	PNT	VAS	+
Couilliot et al. [96]	2013	Chronic pain	Acupuncture	PRSQ	+
Cui et al. [97]	2015	Low back pain	Acupuncture	BPI	+
Davis et al. [98]	2018	Chronic pain	Acupuncture	PROMIS	+
Di Carlo et al. [99]	2021	Fibromyalgia Syndrome	Acupuncture	FAS	+
Persson et al. [100]	2015	Chronic headache	Acupuncture	VAS	+
Sandberg et al. [101]	1998	Fibromyalgia syndrome	Acupuncture	VAS	+
Seroussi et al. [102]	2003	Chronic low back pain	PNT	VAS	+

*Note:* Sleep outcome measures: BPI: Brief Pain Inventory–sleep interference; effect of intervention: (+): positive effect.

Abbreviations: PNT, percutaneous neuromodulation therapy; PRSQ, Pain‐Related Symptoms Questionnaire; FAS, Fibromyalgia Assessment Scale; PROMIS, Patient‐Reported Outcomes Measurement Information System; VAS, Visual Analog Scale

### 3.2. Interventions Reported

The articles included in this scoping review universally utilized a needling intervention that met the inclusion/exclusion criteria. Of the 54 studies included, 36 (67%) investigated acupuncture techniques rather than DN. All 36 acupuncture studies employed traditional Chinese medicine methodology. These were retained because they met the inclusion criteria for needling interventions despite their anatomical targets differing from DN. This distinction is noted to avoid misinterpretation of results. Of the remaining studies, 14 (26%) investigated DN/percutaneous electrical nerve stimulation (PENS), and 4 (7%) investigated percutaneous neuromodulation therapy (PNT).

### 3.3. Sleep Outcome Measurement

Various outcome measurements were used to quantify sleep disturbance in the studies included in this scoping review. Of the 54 articles in this scoping review, 17 used the visual analog scale (VAS), 11 used the Pittsburgh Sleep Quality Index, 4 used Brief Pain Inventory (BPI), 4 used the Numerical Rating Scale (NRS), 5 used the Fibromyalgia Impact Questionnaire (FIQ), 3 used the Insomnia Severity Index (ISI), 2 used the Nottingham Health Profile (NHP)—sleep subgroup, and 8 were single‐group studies.

### 3.4. Needling Effect on Sleep Disturbance

An improvement in sleep disturbance from needling intervention was reported in 41/54 (76%) studies with respect to the outcome measurement tool used. A total of 33 of 41 studies were RCTs with 8/33 with moderate‐to‐high quality (score of 7) [50, 78, 79, 80, 82, 83, 84, 87], and only 5/33 demonstrating scores identified as optimal quality (score ≥ 8/10) [49, 88, 89, 91, 94]. No significant improvement in sleep disturbance was reported in 12/54 (22%) studies, all of which were RCTs, including 3 of moderate‐to‐high quality [81, 85, 86] and 4 of optimal quality [51, 90, 92, 93]. The direct assessment of the needling effect on sleep disturbance was not determinable for one study that had a PEDro score of 5/10 [70]. Overall, studies with optimal quality in this review demonstrate contradictory conclusions, with 5/9 (56%) reporting a positive effect on sleep, but 4/9 (44%) showing no change.

## 4. Discussion

### 4.1. Summary of Evidence

To date, no scoping review has been conducted to explore existing evidence to investigate whether needling intervention affects sleep disturbance in patients with musculoskeletal pain. In this scoping review, 54 primary studies were identified, with 18/54 (33%) studies being moderate‐to‐high quality and 9/54 (17%) being optimal quality. This outcome highlights the paucity of high‐quality research available on this topic of interest, whereas the findings of studies with optimal quality included in this review were mixed.

### 4.2. Sleep Outcome Measurement Reported

Studies with a PEDro score ≥ 7/10 used a variety of sleep outcome measurements to report the DN effects on sleep. The outcome most frequently reported (7/20 studies) [50, 86, 87, 88, 91, 93, 94] was the PSQI. The PSQI has been validated in assessing sleep function in individuals with chronic pain [103, 104]. It is among the most widely used sleep health assessment tools in clinical and nonclinical populations [104]. Its frequent use in sleep research may be because the PSQI addresses essential constructs of sleep, consisting of subjective sleep quality, sleep latency, sleep duration, habitual sleep efficiency, sleep disturbances, use of sleeping medications, and daytime dysfunction over the last month [105, 106].

Apart from two studies [49, 51] in which the Verran Snyder–Halpern Sleep Scale and the ISI were used, most of the remaining 20 studies utilized outcome measurements that could limit their assessment of sleep function. For example, 3 studies [50, 51, 78] used a single‐item question (Likert scale, NRS, and VAS) to measure sleep, 5 studies [82, 83, 85, 90, 92] isolated an individual portion of a quality of life assessment (e.g., BPI, FIQ, and NHP), and 3 studies [79, 84, 89] used tools with limited validity in the assessment of sleep function (e.g., Minor Sleep Evaluation Profile–Sleep Dimension [MSEP], Sleep and Pain Activity Questionnaire [SPAQ], and Visual Analogue Sleep Quality Scale [VASQS]).

The heterogeneity of sleep outcome measures used across the studies may have biased the results of this review. It has been previously reported that when a scoping review encounters a high degree of heterogeneity in outcome measures, accurately synthesizing and comparing findings may be difficult, potentially resulting in misleading conclusions [107]. Furthermore, the lack of uniform use of a validated sleep assessment tool highlights the need for consensus on standardization across research reporting on sleep function to decrease heterogeneity while improving the rigor of evidence.

### 4.3. Needling Effects on Sleep

Despite many studies (41/54) included in this review reporting a positive effect of needling intervention on sleep outcome measurements, information extracted should be interpreted cautiously as 8 of these 41 studies were non‐RCT single‐group clinical trials, and 20/33 RCTs were of low‐to‐moderate quality (PEDro score < 7). The remaining 13 studies [49, 50, 78–80, 82–84, 87–91, 94] reported statistically significant changes (*p* < 0.001) in their respective outcome measures used. Only 6 of these 13 studies utilized validated sleep assessment tools [49, 50, 87, 88, 91, 94].

Although only 22% (12/54) of the included studies reported no significant effect on sleep disturbance, 58% (7/12) of the studies were of moderate‐to‐high quality. Of these studies, 3 utilized validated sleep assessment tools [51, 86, 93], and 2 studies [90, 92] used sleep assessment tools validated for the specific patient populations (i.e., FIQ). The result may suggest that the needling techniques used in these studies (i.e., acupuncture) may not be effective in addressing sleep disorders in certain conditions, such as fibromyalgia and postblunt trauma. Potential mechanisms linking DN to improved sleep include modulation of nociceptive input, reduction of proinflammatory cytokines (IL‐1β and TNF‐α), and increased endogenous opioid release via the endocannabinoid system [32, 33]. These pathways may explain analgesic and sleep‐promoting effects. However, studies reporting no effect may reflect insufficient treatment dosage, heterogeneity in outcome measures, or patient‐specific factors such as central sensitization.

### 4.4. Variety of Treatment and Patient Population

The criteria definition of “dry needling” used for inclusion in this study introduced a variety of treatment interventions that varied not only in technique but also in frequency, dosage, and study design. Despite an eclectic grouping of interventions, results were mixed across all interventions in studies with a PEDro score ≥ 7/10. When patient population is taken into account, chronic spine pain was the most commonly reported condition (*n* = 634) [50, 76, 78, 80, 84, 86, 93], followed by chronic pain disorders (*n* = 863) [49, 82, 88, 89, 90, 92], acute injury (*n* = 130) [51, 81], chronic orofacial pain (*n* = 90) [79], chronic plantar fasciitis (*n* = 90) [94], and carpal tunnel syndrome (*n* = 51) [85].

### 4.5. Implications for Practice, Policy, and Future Research

The results of this scoping review demonstrate the gap in the literature regarding needling intervention and its effect on sleep disturbance, with the findings being mixed. With the increased interest in sleep regarding pain research, incorporating sleep assessment into future study designs should be strongly considered [108]. Furthermore, recent systematic scoping reviews have highlighted the lack of routine sleep measurement for populations with musculoskeletal disorders, such as chronic low back pain [109] and knee osteoarthritis [110]. Despite the robust evidence demonstrating a bidirectional relationship between pain and sleep dysfunction, there continues to be an absence of sleep assessment and measurement in research and the clinical setting.

Overall, the included studies demonstrated substantial variability in terms of intervention techniques, sleep measurement tools, and participant populations. Specifically, the heterogeneity of outcome measures made synthesis and comparison of findings difficult. Implementing strategies for controlling confounding variables may result in more substantial evidence supporting the positive effect of DN on sleep dysfunction such as standardizing sleep assessment tools, needling intervention protocols, and inclusion/exclusion criteria across studies.

### 4.6. Limitations

Studies that were determined eligible were required to be published in English. As many acupuncture articles were published in other languages, such as Chinese, this scoping review was not completely inclusive of all evidence and could affect the comprehensiveness of this review. The needling interventions of interest in this study are commonly identified as complementary and alternative medicine. Such reviews focusing solely on English‐language studies could lead to different results and conclusions than language‐inclusive reviews in this topic area [111]. Furthermore, despite a preliminary grey literature search being performed, these sources were not included in the data synthesis. This may limit the comprehensiveness of the review and should be considered when interpreting findings.

### 4.7. Conclusions

Due to significant variability in treatment intervention, sleep outcome measurement, patient population, and quality of study methodology in the literature, the evidence is mixed and inconclusive to support or refute the effects of DN on sleep deficits in patients with musculoskeletal pain. In addition, it is important for future studies investigating the effects of needling interventions on musculoskeletal pain conditions to include valid sleep outcome measurements.

## Author Contributions

R.M.L.: conceptualization, data curation, formal analysis, investigation, methodology, project administration, writing–original draft, and writing–review and editing (equal). G.T.: conceptualization (supporting), investigation, resources, and writing–review and editing (supporting). S.W‐P.: conceptualization, investigation, methodology, resources, visualization, writing–original draft (supporting), and writing–review and editing (equal).

## Funding

No funding sources were required to complete this scoping review.

## Disclosure

All authors agreed on the final manuscript.

## Ethics Statement

No ethical approvals or patient consent were required for this study.

## Conflicts of Interest

The authors declare no conflicts of interest.

## Data Availability

The data that support the findings of this study are available from the corresponding author upon reasonable request.
